# Nematicidal Screening of Aqueous Extracts from Plants of the Yucatan Peninsula and Ecotoxicity

**DOI:** 10.3390/plants11162138

**Published:** 2022-08-17

**Authors:** Jesús Aviles-Gomez, Jairo Cristóbal-Alejo, María Fé Andrés, Azucena González-Coloma, Germán Carnevali, Daisy Pérez-Brito, Felicia Amalia Moo-Koh, Marcela Gamboa-Angulo

**Affiliations:** 1 Centro de Investigación Científica de Yucatán, Mérida 97205, Mexico; 2 Tecnológico Nacional de México, Campus Conkal, Conkal 97345, Mexico; 3 Instituto de Ciencias Agrarias, CSIC, 28006 Madrid, Spain

**Keywords:** *Alseis yucatanensis*, *Croton itzaeus*, *Helicteres baruensis*, *Meloidogyne incognita*, *Meloidogyne javanica*, nematicidal effects, ecotoxicology

## Abstract

Active metabolites from plants are considered safer than synthetic chemicals for the control of plant-parasitic nematodes of the genus *Meloidogyne*. In the present work, 75 aqueous extracts (AEs) from different vegetative parts of 34 native plant species of the Yucatan Peninsula were evaluated against second-stage juveniles (J2s) of *Meloidogyne incognita* and *M. javanica* in microdilution assays. The highest mortality (M) against both *Meloidogyne* species was produced by the foliar AE from *Alseis yucatanensis* (M ≥ 94%) and *Helicteres baruensis* (M ≥ 77%) at 3% *w*/*v* after 72 h. Other active AEs at 3% were from the leaves of *Croton itzaeus* and stems of *H. baruensis* (M: 87–90%) on *M. javanica* and the stems of *Annona primigenia* and the leaves of *Morella cerifera* on *M. incognita* (M: 92–97%). The AEs from *A. yucatanensis* had the lowest LD_50_ against *M. incognita* (0.36% *w*/*v*), and against *M. javanica* (3.80% *w*/*v*). In an acute ecotoxicity assay of the most promising AEs using non-target earthworms (*Eisenia fetida*), the AE of *A. yucatanensis* had slight acute toxicity (LD_50_: 2.80% *w*/*v*), and the rest of the most active AEs were not ecotoxic. These tropical plants are potential candidates for further studies as biorational agents for controlling *Meloidogyne* species.

## 1. Introduction

Plant-parasitic nematodes cause 8.8–14% of the annual crop losses worldwide, at an estimated cost of approximately USD 173 billion [1,2]. Root-knot nematodes (RKNs) are one of the groups (*Meloidogyne* sp.) with the highest pathogenic capacity and the greatest number of hosts. Of the 105 described species in the genus *Meloidogyne*, *M. arenaria*, *M. hapla, M. incognita,* and *M. javanica* in particular parasitize many important crops [2,3,4]. These RKN species attack the root vascular system, causing water and nutrient transport deficiencies, wilting and chlorosis, retarding plant growth, and reducing yields [5,6]. In addition, they suppress the host defense system, making plants more susceptible to other plant pathogens such as bacteria, fungi, and viruses [7,8].

*Meloidogyne* has been controlled using agronomic practices such as fallow, crop rotation, resistant varieties, and mainly by synthetic chemical nematicides [9]. However, the use of synthetic nematicides such as methyl bromide, 1,3-dichloropropene, carbamates (oxamyl), and organophosphates (fenamiphos), among others, is being restricted because of their high toxicity [10,11]. Requirements for low or no residual synthetic pesticides in the food chain have led to an increased demand to use alternatives with low or no toxicity [9,12]. Plant extracts or their metabolites have been shown to have nematicidal activity [13,14]. For example, the aqueous extracts (AEs) of *Azadirachta indica* [15], *Taxus baccata* [16], and *Xanthium strumarium* [17] cause high mortality to *M. incognita*, and the AEs of *Crambe abyssinica, Cuminum cyminum, Curcuma longa*, *Nigella sativa*, and *Piper nigrum* and have lethal effects against *M. javanica* [18,19].

However, studies that focus on the ecotoxicity of natural pesticides are scarce [20,21]. Earthworms (*Eisenia fetida*) are used as a bioindicator to evaluate the acute and chronic toxicity of synthetic or natural products because they are one of the most common soil species [22]. The assays using these organisms can be indicative of short-term effects and possible long-term damage and thus help to prevent harmful impacts on the environment and humans [20]. For example, the nematicidal hydrolate fraction obtained from *Lavandula luisieri* was slightly toxic to *E. fetida* [21], while fraction F6 from *Couroupita guianensis* was not toxic [23].

Harboring 23,314 species of vascular plants among 2854 genera and 297 families, Mexico occupies fourth place in the world for its floral diversity [24]. However, few of these plant species have been surveyed so far. Of those surveyed, 37 have been detected with nematicidal properties against plant and animal parasitic nematodes [25]. In the continuing search for natural alternatives among the biodiversity of Southern and Southeastern Mexico, a working group has screened 20 species from the region against various phytopathogens. Aqueous extracts (AEs) of the leaves and roots of *Calea urticifolia* and an ethanol extract of the leaves of *Eugenia winzerlingii* had high activity against *Meloidogyne incognita* and/or *M. javanica* [26,27,28]. Such bioprospecting for natural nematicidal agents must therefore be intensified.

In the present research, we evaluated AEs from 34 plant species from the Yucatan Peninsula (Table 1) against second-stage juveniles (J2s) of *M. incognita* and *M. javanica*. The most effective AEs against both nematodes were assayed against adults of the earthworm *E. fetida* and in a serial dilution to calculate their median and 90 lethal doses against the targets.

## 2. Results

### 2.1. Nematicidal Activity of Aqueous Extracts

Among 75 AEs, from different plant parts of 34 plant species, tested against the J2s of *M. incognita* at 6% (*w/v*) concentration, 28 induced high mortality (≥90%) at 72 h of exposure (Table 2). Among these, eight AEs were lethal (M of 100%) against *M. incognita*: *Alseis yucatanensis* leaves (L), *Annona primigenia* stem (S), *Hybantus yucatanensis* (L, S), *Macroscepis diademata* (S), *Randia aculeata* (L, roots (R)), and the entire plant of *Calea jamaicensis.* At 3% (*w*/*v*), 17 AEs caused mortality >75% against *M. incognita.* These active AEs included *A. yucatanensis* (L), *Alvaradoa amorphoides* (R), *A. primigenia* (S), *Chrysophylun mexicanum* (R), *Diospyros* sp. (L), *Eugenia* sp. (L), *Helicteres baruensis* (L), *H. yucatanensis* (S), *M. diademata* (L), *Malpighia glabra* (R), *Morella cerifera,* (L), *Piper neesianum* (L, R)*, Randia aculeata* (L), and the complete plant of *C. jamaicensis, Euphorbia armourii*, and *Ipomoea clavata* (Table 2).

In the nematicidal evaluation of the AEs at 6% (*w/v*) against the J2s of *M. javanica*, one plant part from seven plant species and two plant parts from another plant caused mortality ≥90% within 72 h (Table 2). These AEs were from *A. yucatanensis* (L), *C. latifolia* (S, R), *Croton arboreus* (S), *C. itzaeus* (S), *Diospyros* sp. (L), *H. baruensis* (S)*, M. glabra* (L), and *Tabernaemontana donnell-smithii* (L). When these nine highly toxic AEs from eight different plant species were tested at 3% (*w/v*), five caused high mortality (>75%): AEs from the leaves of *A. yucatanensis, C. itzaeus, C. latifolia,* and *H. baruensis* and from the stems of *H. baruensis* (Table 2).

**Figure 1:**
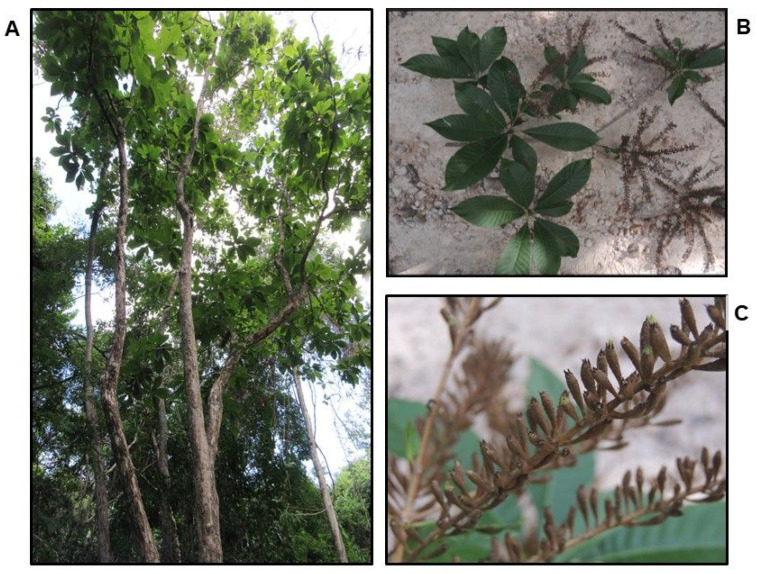
*Alseis yucatanensis*: (**A**) tree; (**B**) leaves; (**C**) inflorescence.

**Figure 2:**
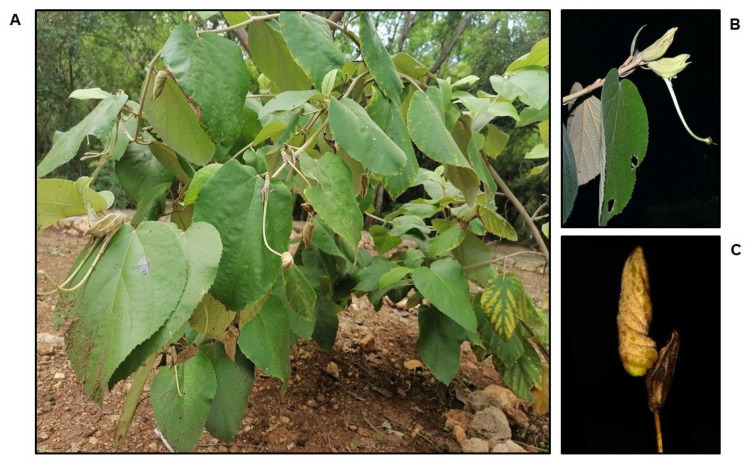
*Helicteres baruensis*: (**A**) shrub; (**B**) flower; (**C**) fruit.

In general, the AEs from the leaves of *A. yucatanensis* and *H. baruensis* were the most active against both nematodes. The AE of *A. yucatanensis* had the lowest LD_50_ against both species, 0.36% (*w/v*) on *M. incognita* and 3.80% *w/v* on *M. javanica,* and the lowest LD_90_ (1.83% *w/v*) against *M. incognita.* Interestingly, the LD_90_ was very similar for the AEs of *A. yucatanensis* and *H. baruensis* against *M. javanica*, 5.64 and 5.71% (*w/v*), respectively. In addition, there was a significant difference in the LD_50_ and LD_90_ between the two plant species against *M. incognita* but not for *M. javanica* (Table 3, Figure 1 and Figure 2).

### 2.2. Ecotoxicity of the AE of Alseis yucatanensis on Eisenia fetida

**Figure 3:**
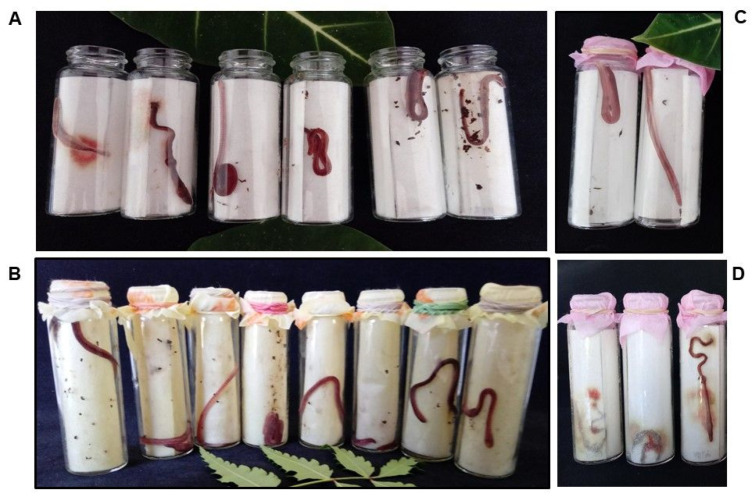
Acute contact ecotoxicity assay of (**A**) *Alseis yucatanensis* (1 mL, 3% *w*/*v*); (**B**) *Helicteres baruensis* (1 mL, 6% *w*/*v*), (**C**) water (1 mL), (**D**) oxamyl (1 µL/mL) using filter paper (49.92 cm^2^) and *Eisenia fetida*.

The AE from the leaves of *A. yucatanensis* had an ecotoxicological effect within 72 h on adults of *E. fetida* at 3% (*w*/*v*) and was lethal at 6% (*w*/*v*). Treatments with serial dilutions of this AE showed that the LD_50_ was 2.80% (*w*/*v*) and the LD_90_ was 4.72% (*w*/*v*) (Table 4). By contrast, the AE of *H. baruensis*, and other most active AEs from leaves of *A. primigenia*, *C. itzaeus* and *M. cerifera*, were not ecotoxic to adults of *E. fetida* in this acute assay (Figure 3).

## 3. Discussion

The present contribution is part of the first bioprospecting research on native plant extracts against *M. javanica* and the second against *M. incognita*. Further, it enriches our knowledge about the natural nematicidal properties of some plant species of the flora of the Yucatan Peninsula. Previous studies on 55 plant extracts from 20 native plants showed that the AE from *C. urticifolia* (2.6%, *w*/*v*) and ethanol extracts from *C. urticifolia, E. winzerlingii,* and *Tephrosia cinerea* (500 ppm) were effective against *M. incognita* [26,27,28]. We found no reports of nematicidal activity for the plant species studied here after an exhaustive literature search. Bioprospecting of the 75 AEs from native plant species for activity against the J2s of *M. javanica* and *M. incognita* led to the detection of 33 AEs (44%) from 20 plant species (59% of the total) with high effectiveness (M = 90–100%) at 6 *w/v*. At 3% (*w*/*v*), 22 AEs (20% of the total) caused >75% mortality against at least one nematode species. 

The J2s of *M. incognita* were more sensitive to plant extracts; 19 AEs (25% of the total tested) caused >75% mortality at 3%. Other authors reported similar mortality to what we found, but the AE concentrations were higher. For example, AEs from *Achyranthes aspera, Ageratum conyzoides,* and *Solanum xanthocarpum* leaves at 33% (*w*/*v*) caused 84–98% mortality after 48 h of exposure to J2 larvae of *M. incognita* [29,30]. AEs from *C. longa, N. sativa,* and *P. nigrum* caused 36% mortality against the J2s of *M. javanica* at 40% (*w/v*) after 72 h [18]. Likewise, AEs from the seeds and shoots of *A. indica* and leaves of *Nerium oleander* and *Olea europea* caused 53–65% mortality at 10% (*w/v*) after 48 h [31]. The AEs tested here induced higher nematicidal activity against *M. incognita* J2s at lower concentrations. Furthermore, the LD_50_ and LD_90_ of the AE from the leaves of *A. yucatanensis* against the J2s of *M. javanica* after 72 h were lower than those documented for the AEs from the leaves of *Cinnamomum zeylanicum* (LD_50_ of 15.38 mg/mL and LD_90_ of 24.73 mg/mL) and *Eugenia caryophyllata* (LD_50_ = 17.91 mg/mL and LD_90_ = 23.45 mg/mL) after 24 h [32]. AEs obtained with water extraction at 100 °C, for 10 h and 14 h at 25 ºC, from *C. abyssinica* at 1:10 (*w*/*v*) were reported to cause 72.2% mortality in J2s of *M. javanica* after 24 h [19]. In particular, more AEs have been tested against *M. incognita* than against *M. javanica* based on our search of the literature. Reports of plant extracts tested against *M. javanica* more commonly focused on organic extracts and essential oils [14]. On the other hand, the nematicidal potential of hydrolate distilled waters that remain after hydro- or steam distillation, and separation of the essential oil, have been amply demonstrated [33]. Specifically, the nematicidal effects in vitro and in vivo of hydrolates from Spanish aromatic plant species *Alium sativum*, *Artemisia absintium*, *Lavandula* × *intermedia* var. Super, *L. luisieri, Thymus vulgaris, T. zygis*, and purple garlic have been tested against *M. javanica* [34,35,36,37]. Furthermore, hydrolates from Greek aromatic species *Origanum vulgare*, *Mentha piperita,* and *Melissa officinalis*, *Satureja hellenica*, and *C. cyminum* showed in vitro high activity towards *M. incognita* and *M. javanica* [38,39,40]. 

In the present work, 25% of the AEs had greater lethality against J2s at 3% *w*/*v* than 6% *w/v*, but the difference in lethality was significant for only eight of these AEs (>12% difference): AEs from *Alvaradoa amorphoides* leaves and roots, *Bakeridesia notolophium* leaves, *Byrsonima bucidifolia* leaves, *Licaria* sp. leaves, *M. cerifera* stem, *P. cubana* stem bark, and *Paullinia* sp. leaves. This phenomenon of lower concentrations causing greater mortality than higher ones has been reported previously. For example, leaf extracts from *C. urticifolia* at 500 ppm caused 90% mortality, but 250 ppm caused 97% mortality against J2s of *M. incognita* [26]. Similarly, the extracts of *Achillea wilhelmsii* at 6% (*w/v*) caused 10.6% mortality, and 15.6% at 3% (*w*/*v*) [41]. These data could be due to pipetting errors, temperature variations, or variations in the extract dosage and exposure duration [42]. Moreover, during incubation, precipitates can be formed in the 6% concentration and adhere to the microplate walls, lowering the concentration of exposure.

The present contribution adds to the few works on the in vitro activity of plant extracts against *M. javanica* and *M. incognita*. For example, organic extracts and fractions of *Eugenia winzerlingii* leaves caused 100% mortality at 1 µg/µL against J2s of *M. javanica* and *M. incognita* within 72 h; such activity was conferred by decanoic, undecanoic, and dodecanoic acids [28]. The essential oils of *S. hellenica* caused 100% paralysis of the J2s of *M. javanica* and *M. incognita* at 200 µL/L after 96 h due to the activity of *p*-cymene and carvacrol [39]. 

Our nematicidal bioassays indicate that the AEs from the leaves of *A. yucatanensis* and *H. baruensis* are the most promising against *M. incognita* and *M. javanica*. Among the 22 plant species belonging to the genus *Alseis* (Rubiaceae), only two are described for Mexico, *A. yucatanensis* and *A. hondurensis* Standl. Both tree species are endemic to Southern Mexico and part of Central America [24,43]. Our report is the first to show the nematicidal activity of an extract from *A. yucatanensis* against J2s of *M. incognita* and *M. javanica*. Studies by the working group have documented that the AE of the leaves of *A. yucatanensis* had no antifungal activity at 2000 µg/mL against the phytopathogens *Fusarium equiseti* and *F. oxysporum* [44]. The only reported activity of this species is the vasorelaxation (VR) of aortic tissue in rats with a median effective dose of 0.12 mg/mL of the AE from the bark [45]. For other species of the genus, ethanol and acetone–water (7:3) extracts of the leaves of *Alseis blackiana* were reported to have moderate in vitro antiviral activity (VR of 0.5 to 1.0) against HSV-1 and HSV-2 (herpes simplex virus) at 100 µg/mL [46]. Nematicidal activity of some Rubiaceae species has been reported: soil amendments at 1.0% (*w/w*) of plant debris from *Coffea arabiga* reduced the incidence of root galls on *Cucurbita pepo* caused by *M. arenaria*, and an aqueous extract of *Moringa pterygosperma* leaves at 1:5 caused 100% mortality of the J2s of *M. incognita* after 24 h [47]. However, nothing is known about the phytochemistry of *A. yucatanensis*. In the genus *Alseis*, only alkaloid components from *Alseis blackiana* were detected by a positive Dragendorff’s test [48]. Indole alkaloids have been widely reported from members of the families *Rubiaceae, Apocynaceae*, and *Loganiaceae*, which have prominent activity against diverse biological targets [49]. The application of powdered leaves (1, 3, and 5 g) of *Catharanthus roseus* is highly effective (M = 71.8, 71.6, and 72.6%) in controlling *M. incognita* infection in potted pumpkins (*Cucumis sativum*) [50]. Waltheriones E and A from *Triumfetta grandidens* are effective against *M. incognita* [51], and harmine from *Peganum harmala* is active against *M. javanica* [52].

The genus *Helicteres* (Malvaceae) consists of 60 species distributed mainly in America, with four species described from Mexico and three of them, *H. baruensis*, *H. guazumifolia*, and *H. mexicana*, from the Yucatan Peninsula [53,54]. This genus is found in tropical and subtropical regions, mainly in deciduous forests. The plant *H. baruensis* (Mayan name of *sutup)* is first reported with nematicidal activity for the species and genus. Petroleum ether and chloroform extracts from *H. baruensis* have action against *Salmonella enteritidis* and *Bacillus cereus*; this activity is attributed to sterols and alkaloids [55], but their ethanol and AE did not have antifungal activity against *F. equiseti* or *F. oxysporum* [44]. Further, their methanol and dichloromethane extracts had no anti-neoplastic activity against the LNCaP prostate cancer cell line [56]. In addition to sterols and alkaloids, polyphenols are also present in its leaves [57]. 

Earthworms have been used for in vitro ecotoxicity assays of pesticides to evaluate environmental risk. The filter paper contact test is used to determine the initial acute toxicity of soil contaminants [22]. In the present study, the ecotoxicity of *A. yucatanensis* caused slight acute toxicity towards *E. fetida*. Other studies on the AE, such as artificial soil tests [22] and chronic ecotoxicity tests, should be conducted next. These toxicity tests have not been commonly reported for natural extracts or metabolites from plants or other organisms [21]. Among these, an essential oil from *Piper betle* at 1000 µg/cm^2^ was innocuous to *E. fetida*; mortality was only 5.4% after 48 h in the filter paper contact test [58]. Cucurbitacin E from *Citrus colocynthis* had good efficacy after 48 h against *Spodoptera litura* with LD_50_ of 15.84 ppm and LD_90_ of 67.70 ppm, and at 100 ppm, it induced 11% mortality of *E. fetida* [59], corroborating its lack of effect against this non-target soil organism.

After application, the plant extracts readily decompose when exposed to environmental factors such as light and temperature [60]. Therefore, the nematicidal extracts from *A. yucatanensis* and *H. baruensis* and other promising AEs should be further tested in the greenhouse and field for toxic or beneficial effects on plants. The active compounds also need to be identified, purified, and tested for toxicity against additional non-target organisms.

## 4. Materials and Methods

### 4.1. Plant Material

Plant species were collected from 2016 to 2018 in the three states in the Yucatan Peninsula: (1) Xmaben, Hopelchen, Campeche (19°15′42.92″ N, 89°21′45.91″ W); (2) the Jahuactal of Ejido Caobas, Othón P. Blanco (18°15′34″ N, 88°57′14″ W), (3) Punta Pulticub, Othón P. Blanco (19°04′29.96″ N, 87°33′17.15″ W), and (4) Punta Laguna, Carretera Cobá—Nuevo Xcan (20°38′49.4″ N, 87 °38′02.2″ W) in Quintana Roo; (5) Kaxil Kiuic, Oxkutzcab, Yucatan (20° 06′10.8″ N; 89°33′43.2″ W). The leaves, stem, stem bark, roots, and root bark were collected from most plant species, and complete plants were collected for some species. One specimen of each collected species was deposited in the Herbarium-Fibroteca *U Najil Tikin Xiw*”of the Centro de Investigación Científica de Yucatán (Table 1 and Table 2). 

The plant material was dried in a lamp chamber (50–60 °C) for 3 days and crushed in a grinder (Model 1520, Pagani, Azcapotzalco, Mexico) with blades (5 mm screen) and kept in the dark in a cold room until use. A total of 75 samples from the 34 plant species were obtained.

### 4.2. Preparation of Aqueous Extracts (AEs)

The powdered plant material (1.5 g) was added to 20 mL of boiling distilled water for 5 min, then cooled and filtered through cheesecloth cotton and filter paper (Whatman No. 1). The water volume was brought to 25 mL to obtain an AE at an initial concentration of 6% *(w/v)*. All AEs were frozen (−17.5 ± 0.5 °C) and lyophilized (Labconco FreeZone 2.5, model 7670520, Houston, TX, USA) for 15 h to reduce the volume to 50% and obtain a concentration of 12% *(w*/*v)* of the original plant material for each extract. The concentrated AEs were then sterilized using a 0.22 µm Millipore filter (Merck-Millipore, Burlington, MA, USA) and frozen until use.

### 4.3. Nematicide Bioassay 

The nematode populations of *M. javanica* were obtained from the Instituto de Ciencias Agrarias (25 ± 1 °C/70% relative humidity (RH)), and *M. incognita* from the Tecnológico Nacional de México—Campus Conkal (30 ± 2 °C/90% RH). The populations of both species were maintained on *Solanum lycopersicum* (var. Marmande) plants in plastic pots in a greenhouse. For bioassays, egg masses were manually recovered from galled roots and incubated for 72 h in sterile water at 30 ± 2 °C for *M. incognita* and 25 ± 1 °C for *M. javanica*. Hatched second-instar juveniles (J2s) were adjusted to a final concentration of 100 J2s/100 μL of distilled water [14,26].

Each well of a 96-well U-bottom plate (NEST Biotechnology, Wuxi, Jiangsu, China) received 100 µL of the AE (12%, *w*/*v*) and 100 µL of the nematode suspension. As a positive control, the synthetic nematicide Vydate^®^ (oxamyl 235 g/L) was used at 1 µL/mL, and the suspension of nematodes in distilled water (100 J2s/100 µL) was used as a negative control. For each treatment, four replicate wells were evaluated. The experimental plates were sealed and incubated in the dark using the same conditions used for the egg masses (vide supra) described by Moo-Koh et al. [14,61].

Any immobile J2 larvae that were insensitive to a needle touch were counted as dead at 72 h and expressed as percentage mortality (M%). The data obtained from the nematicidal activity are presented as corrected percentages of J2s mortality (Schneide–Orelli formula: % efficiency = (*b* − *k*/100 − *k*) × 100, where *b* = % of dead individuals in the treatment and *k* = % of dead individuals in the control [62]. The extracts that caused the greatest mortality were then evaluated at serial dilutions (6, 3, 1.5% *w/v* or less). The LD_50_ and LD_90_ values were determined using a probit analysis with SAS ver. 9.4 for Windows (SAS Institute, Cary, NC, USA).

### 4.4. Eisenia Fetida Assay 

For the *E. fetida* assay (a contact test on filter paper, OECD 207 1984), 1 mL of the test AE at 6 and 3% (*w/v*) was dropped onto a Whatman No. 1 filter paper (6.4 cm long × 7.8 cm wide) at a final concentration of 1200 and 600 µg/cm^2^ and kept in a hood extractor until completely dry. The filter paper was then placed in a glass vial (8 cm long × 3 cm in diameter). The earthworms were purchased (PlanetaMexico, Mexico City, Mexico) and maintained in organic horse manure and soil at 28 ± 2 °C until they were adults (2 months old) and weighing 300–600 mg each. Then, they were washed with water and kept on moist filter paper in the dark for 3 h to ensure that all intestinal contents had been released. Water was used as a negative control and a synthetic nematicide (Vydate^®^ (oxamyl 42%), Dupont) as a positive control at 1 µg/mL (0.02 μg/cm^2^). Each vial received 1 mL of distilled water to keep the filter paper moist and one earthworm deposited; then, vials were covered with a gauze-type cloth that was secured with a rubber band to ensure ventilation and retain the earthworms in the vial. For each treatment, 20 replicate vials were used. The vials were placed in a humid chamber at 26 ± 2 °C in the dark. Every 24 h for 72 h, the worms were checked and considered dead if they did not respond to a small stimulus in the anterior region. Any pathological behaviors or symptoms were also recorded [22].

Serial dilutions (6, 3, 1.5% *w/v* or less) of the AE with ecotoxicity were tested and worms checked every 24 h for 72 h as described above. The results were processed in SAS software (SAS Institute, Cary, NC, USA) to obtain the LD_50_ and LD_90_.

## 5. Conclusions

Our knowledge of Mexican flora as potential nematicides has been enriched. In this approach to surveying plant species in the Yucatan Peninsula for bioactivity against phytopathogenic nematodes, we confirmed that native species caused high mortality against the J2s of the phytonematodes *M. incognita* and *M. javanica*. The AEs from *A. yucatanensis* and *H. baruensis* leaves were the most effective; the first had low ecotoxicity, and the second none. With high efficacy in vitro against both nematodes, the aqueous extracts of *A. yucatanensis* and *H. baruensis* are highly promising candidates for developing products to control *Meloidogyne* nematodes.

## Figures and Tables

**Table 1:** Native plants from the Yucatan Peninsula evaluated against second-stage juveniles of *Meloidogyne incognita* and *M. javanica*.

Plant Species	Family	Site	Voucher
*Alseis yucatanensis* Standl.	Rubiaceae	5	JLT-3179
*Alvaradoa amorphoides* Liebm.	Simaroubaceae	2	GC 8236
*Annona primigenia* Standl. & Steyerm.	Annonaceae	2	GC-8057
*Bakeridesia notolophium* (A. Gray) Hochr.	Malvaceae	3	RD-s/n
*Byrsonima bucidifolia* Standl.	Malpighiaceae	2	GC-8087
*Calea jamaicensis* (L.) L.	Asteraceae	2	GC-8084
*Cameraria latifolia* L.	Apocynaceae	2	JLT-1165
*Chrysophyllum mexicanum* Brandegee ex Standl.	Sapotaceae	2	GC-8082
*Coccoloba* sp.	Polygonaceae	1	GC-8258
*Croton arboreus* Millsp.	Euphorbiaceae	2	JLT-1132
*Croton itzaeus* Lundell	Euphorbiaceae	2	JLT-1138
*Diospyros* sp.	Ebenaceae	4	GC-8147
*Erythroxylum confusum* Britton	Erythroxylaceae	2	GC-8179
*Eugenia* sp.	Myrtaceae	4	GC-8127
*Euphorbia armourii* Millsp.	Euphorbiaceae	5	JLT-3182
*Guettarda combsii* Urb.	Rubiaceae	2	GC-8047
*Helicteres baruensis* Jacq.	Malvaceae	5	GC-8127
*Hybanthus yucatanensis* Millsp.	Violaceae	4	GC-8158
*Ipomoea clavata* (G. Don) Ooststr. ex J.F. Macbr	Convolvulaceae	5	JLT-3181
*Karwinskia humboldtiana*(Willd. ex Roem. & Schult.) Zucc.	Rhamnaceae	5	JLT-3188
*Licaria* sp.	Lauraceae	2	GC-8037
*Macroscepis diademata* (Ker Gawl.) W.D. Stevens	Apocynaceae	5	JLT-3187
*Malpighia glabra* L.	Malpighiaceae	4	GC-8144
*Morella cerifera* (L.) Small.	Myricaceae	2	JLT-1137
*Mosannona depressa* (Baill.) Chatrou	Annonaceae	2	GC-8085
*Parathesis cubana* (A. DC.) Molinet & M. Gómez	Primulaceae	2	JLT-1133
*Paullinia* sp.	Sapindaceae	4	GC-8106
*Piper neesianum* C. DC.	Piperaceae	2	GC-8080
*Psychotria* sp.	Rubiaceae	2	GC-8086
*Randia aculeata* L.	Rubiaceae	4	GC-8156
*Serjania caracasana* (Jacq.) Willd	Sapindaceae	4	GC-8114
*Simarouba glauca* DC.	Simaroubaceae	2	GC-8081
*Tabernaemontana donnell-smithii* Rose	Apocynaceae	2	GC-8056
*Turnera aromatica* Arbo	Passifloraceae	2	GC-8081

—Sites: (1) Xmaben, Campeche; (2) Ejido Jahuactal; (3) Punta Pulticub; (4) Punta Laguna, Quintana Roo; (5) Kaxil Kiuic, Yucatan.

**Table 2:** Nematicidal effects of aqueous extracts from native plants of the Yucatan Peninsula against second-stage juveniles (J2) of *Meloidogyne incognita* and *M*. *javanica* after 72 h of exposure.

Plant Species	Plant Part ^a^	J2 Mortality (%) ^c^			
		*Meloidogyne incognita*		*Meloidogyne javanica*	
		6% (*w/v*) ^b^	3% (*w/v*) ^b^	6% (*w/v*) ^b^	3% (*w/v*) ^b^
*Alseis yucatanensis*	L	100 ± 0.00	94 ± 1.01	100 ± 0.00	100 ± 0.00
*Alvaradoa amorphoides*	L	33 ± 0.91	74 ± 1.17	9 ± 2.34	32 ± 22.68
	S	29 ± 0.24	24 ± 2.54	15 ± 2.44	13 ± 3.06
	R	30 ± 0.43	76 ± 2.57	34 ± 22.07	11 ± 0.72
*Annona primigenia*	L	94 ± 0.78	71 ± 7.33	74 ± 11.46	75 ± 4.28
	S	100 ± 0.00	97 ± 1.83	51 ± 22.57	44 ± 12.46
*Bakeridesia notolophium*	L	23 ± 0.38	66 ± 1.39	2 ± 1.03	6 ± 2.60
	S	4 ± 1.32	6 ± 0.16	28 ± 23.96	14 ± 5.82
*Byrsonima bucidifolia*	L	60 ± 3.45	17 ± 1.29	2 ± 0.83	24 ± 20.18
	S	83 ± 0.73	22 ± 2.59	15 ± 1.62	5 ± 0.60
	R	81 ± 0.39	22 ± 1.38	6 ± 1.50	11 ± 1.83
*Calea jamaicensis*	WP	100 ± 0.44	83 ± 1.02	89 ± 3.03	18 ± 2.61
*Cameraria latifolia*	L	94 ± 0.47	71 ± 0.67	81 ± 2.58	80 ± 13.13
	S	ne	30 ± 3.16	94 ± 2.29	8 ± 1.84
	R	88 ± 0.59	74 ± 1.24	94 ± 1.40	9 ± 2.69
*Chrysophylum mexicanum*	L	88 ± 0.48	68 ± 2.55	18 ± 4.08	19 ± 3.40
	S	88 ± 0.42	64 ± 1.26	6 ± 2.06	18 ± 8.66
	R	92 ± 1.07	86 ± 0.77	11 ± 1.68	19 ± 1.28
*Coccoloba* sp.	L	97 ± 0.50	13 ± 0.82	8 ± 1.93	9 ± 18.20
	S	2 ± 0.89	16 ± 0.52	33 ± 1.66	5 ± 1.49
*Croton arboreus*	L	84 ± 0.50	75 ± 1.31	72 ± 2.26	75 ± 4.61
	S	94 ± 1.66	23 ± 0.93	90 ± 2.33	17 ± 6.57
	R	71 ± 4.14	66 ± 2.47	70 ± 10.70	8 ± 1.30
*Croton itzaeus*	L	78 ± 0.26	35 ± 0.58	80 ± 5.73	90 ± 4.01
	S	92 ± 0.49	19 ± 1.23	94 ± 0.54	3 ± 2.69
	R	87 ± 0.31	19 ± 2.63	3 ± 2.28	3 ± 0.99
*Diospyros* sp.	L	87 ± 0.55	84 ± 0.61	85 ± 5.08	5 ± 0.64
*Erythroxylum confusum*	L	86 ± 0.29	36 ± 0.412	4 ± 3.89	6 ± 0.89
*Eugenia* sp.	L	76 ± 0.56	83 ± 0.49	3 ± 1.05	2 ± 1.95
	S	99 ± 0.78	8 ± 3.11	38 ± 1.93	9 ± 3.10
	R	93 ± 3.42	4 ± 1.41	90 ± 2.53	17 ± 4.54
*Euphorbia armourii*	WP	76 ± 0.17	87 ± 0.86	35 ± 9.77	35 ± 17.57
*Guettarda combsii*	L	94 ± 1.37	68 ± 2.93	26 ± 5.08	5 ± 2.52
	S	88 ± 0.53	13 ± 3.59	4 ± 0.59	2 ± 1.63
	R	81 ± 0.52	63 ± 7.89	79 ± 7.50	70 ± 2.38
*Helicteres baruensis*	L	80 ± 1.02	77 ± 5.83	89 ± 0.34	83 ± 2.32
	S	84 ± 0.71	63 ± 1.93	92 ± 2.30	87 ± 2.23
	R	66 ± 1.23	23 ± 2.21	47 ± 18.92	4 ± 1.70
*Hybanthus yucatanensis*	L	100 ± 0.00	74 ± 2.70	43 ± 9.86	5 ± 1.98
	S	100 ± 0.00	88 ± 0.60	55 ± 17.36	3 ± 1.75
*Ipomoea clavata*	WP	83 ± 0.58	88 ± 0.80	19 ± 3.17	9 ± 4.21
*Karwinskia humboldtiana*	L	84 ± 0.58	77 ± 1.72	80 ± 1.25	39 ± 9.19
*Licaria* sp.	L	76 ± 0.33	29 ± 1.12	21 ± 13.41	68 ± 3.46
	SB	89 ± 1.37	24 ± 2.59	60 ± 11.94	24 ± 10.51
	RB	57 ± 0.17	3 ± 1.48	50 ± 6.37	27 ± 4.60
*Macroscepis diademata*	L	94 ± 2.29	88 ± 1.32	86 ± 5.86	25 ± 24.22
	S	100 ± 0.00	73 ± 3.43	34 ± 22.88	5 ± 1.15
*Malpighia glabra*	L	88 ± 2.00	75 ± 0.70	100 ± 0.00	55 ± 20.60
	S	93 ± 2.19	65 ± 4.34	21 ± 7.82	0 ± 0.48
	R	96 ± 0.16	80 ± 1.01	20 ± 8.70	11 ± 6.97
*Morella cerifera*	L	96 ± 0.24	92 ± 0.96	41 ± 20.93	23 ± 18.85
	S	26 ± 0.26	76 ± 3.89	5 ± 1.27	23 ± 3.72
	RB	81 ± 0.31	63 ± 1.63	6 ± 1.30	11 ± 1.14
*Mosannona depressa*	L	90 ± 0.41	67 ± 2.37	63 ± 10.78	40 ± 1.83
	SB	91 ± 0.42	59 ± 3.17	23 ± 2.65	13 ± 3.18
	RB	ne	62 ± 1.14	8 ± 1.57	8 ± 0.46
*Parathesis cubana*	L	88 ± 0.66	52 ± 6.80	2 ± 1.20	4 ± 0.80
	SB	27 ± 4.21	66 ± 3.25	3 ± 0.91	5 ± 1.15
	RB	75 ± 0.44	39 ± 1.89	3 ± 1.47	7 ± 1.47
*Paullinia* sp.	L	40 ± 3.30	5 ± 0.75	3 ± 0.63	31 ± 15.66
	R	22 ± 5.20	5 ± 0.75	7 ± 0.95	2 ± 0.36
*Piper neesianum*	L	90 ± 0.66	78 ± 0.56	ne	ne
	S	92 ± 0.30	74 ± 0.56	41 ± 16.30	50 ± 22.04
	R	92 ± 0.47	84 ± 0.41	31 ± 17.19	8 ± 1.18
*Psychotria* sp.	WP	92 ± 0.48	61 ± 6.52	6 ± 0.74	14 ± 2.04
*Randia aculeata*	L	100 ± 0.00	87 ± 2.26	47 ± 7.14	31 ± 17.53
	S	99 ± 0.82	11 ± 0.53	14 ± 5.43	3 ± 1.12
	R	100 ± 0.00	34 ± 2.41	11 ± 2.76	6 ± 1.26
*Serjania caracasana*	L	26 ± 1.08	5 ± 1.40	6 ± 0.67	4 ± 0.41
*Simarouba glauca*	L	77 ± 0.69	55 ± 5.31	5 ± 1.06	9 ± 0.34
	S	82 ± 1.84	58 ± 1.08	69 ± 14.57	5 ± 1.16
	R	84 ± 0.60	43 ± 2.66	53 ± 17.07	9 ± 0.77
*Tabernaemontana donnell-smithii*	L	86 ± 1.13	23 ± 2.17	90 ± 2.16	3 ± 0.40
	SB	52 ± 0.21	30 ± 5.33	3 ± 4.21	6 ± 0.66
*Turnera aromatica*	WP	58 ± 14.88	51 ± 0.85	27 ± 20.84	8 ± 0.81
Negative control: water		00 ± 0	00 ± 0	00 ± 0	00 ± 0
Positive control: oxamyl		100 ± 0	100 ± 0	100 ± 0	100 ± 0

^a^ Plant parts: leaves (L); stem (S); root (R); root bark (RB); whole plant (WP); not evaluated (ne). ^b^ AE concentrations (*w/v*). ^c^ Values are mean ± standard error of four replicates.

**Table 3:** Lethal doses (50 and 90) of the active aqueous extracts on second-stage juveniles of *Meloidogyne incognita* and *M. javanica.*

Plant Species	*Meloidogyne incognita*		*Meloidogyne javanica*	
	LD_50_ (95% CL) ^a^	LD_90_ (95% CL)	LD_50_ (95% CL)	LD_90_ (95% CL)
*Alseis yucatanensis*	0.36 (0.29, 0.43)	1.83 (1.72, 1.97)	3.80 (3.72, 3.89)	5.64 (5.49, 5.79)
*Helicteres baruensis*	1.34 (1.00, 1.63)	7.36 (6.73, 8.17)	4.05 (3.95, 4.14)	5.71 (5.57, 5.87)

^a^ At least four dilutions (*w/v*) were used, at 72 h, to obtain LD_50_ and LD_90_. CL denotes confidence limit.

**Table 4:** Ecotoxicity and lethal doses (50 and 90) of nematicide aqueous extracts (leaves) against *Eisenia fetida* (one/vial, 20 replicates), after 72 h exposure.

Plant Species	Conc. % (*w/v*)	Ecotoxicity (% Mortality/h) ^a^			Lethal Doses % (*w/v)*	
		24 h	48 h	72 h	LD_50_ (95% CL) ^b^	LD_90_ (95% CL)
*Alseis yucatanensis*	6	10	85	100	2.80 (2.3, 3.53)	4.72 (3.90, 6.30)
	3	5	30	53		
	1.5	5	10	26		
	0.75	0	0	0.5		
*Helicteres baruensis*	6	0	0	0		
	3	0	0	0		
*Annona primigenia*	6	0	0	0		
	3	0	0	0		
*Croton itzaeus*	6	0	0	0		
	3	0	0	0		
*Morella cerifera*	6	0	0	0		
	3	0	0	0		
Negative control: water		0	0	0		
Positive control: oxamyl		100	100	100		

^a^ Corrected mortality percentages; ^b^ at least four dilutions (*w/v*) were used, at 72 h, to obtain LD_50_ and LD_90_. CL denotes confidence limit.

## Data Availability

Not applicable.
